# Supplementary material for: The Origin(s) of LUCA: Computer Simulation of a New Theory
Source: Life (Basel). 2025 Jan 10;15(1):75. doi: 10.3390/life15010075 (PMC11766493; doi:10.3390/life15010075)
Supplement: Supplementary file 1 [file life-15-00075-s001.zip › life-3270335-supplementary.pdf]

Note: In all simulations with GAMA, 1 "cycle" = 1 wet phase or 1 dry phase, alternated.

Figure S-1. Snapshots of four simulations for the evolution of FUCAs (Stage 1). The left window of the panels (A, B, C, D) records the evolutionary process of FUCAs (or type V6 vesicles) in simulation, with the red line highlights the appearance individual FUCAs. The center window plots the number of "cycles" (x-axis) against the number of different types of vesicles (y-axis) within the pond as the system evolves, with the total number of FUCAs in red. The right window summarizes key indicators, including the numbers of alive vesicles (V1-V6), of a specific simulation. See also Table 5 in the main text.

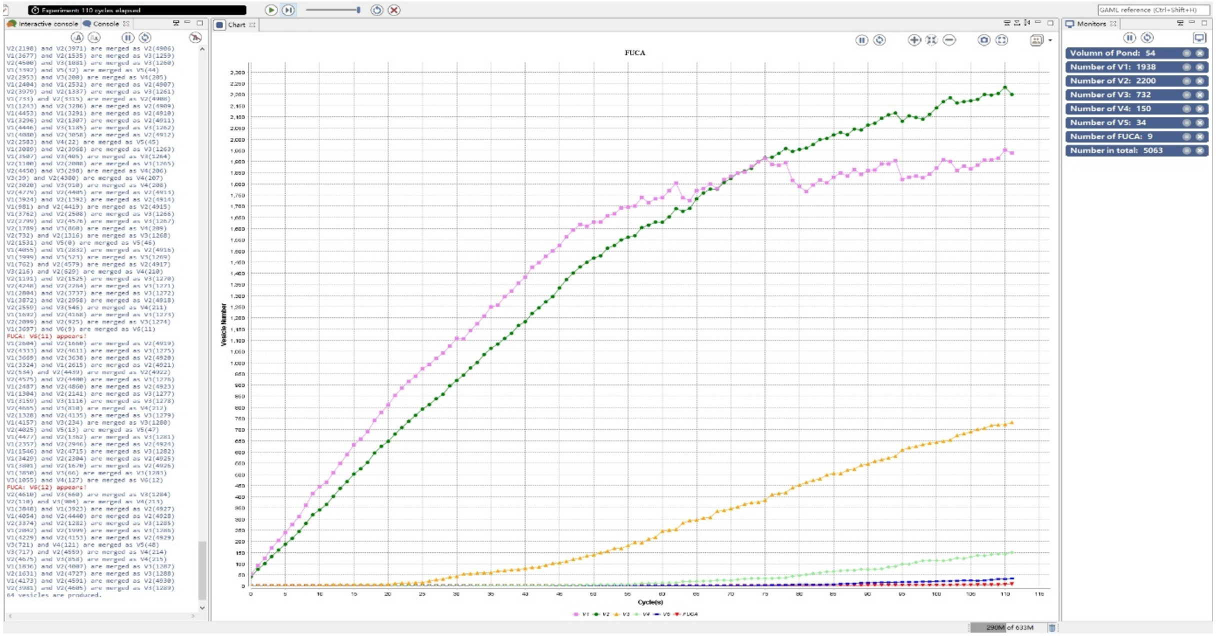

Figure S-1A. 26 FUCAs produced, 112 "cycles"

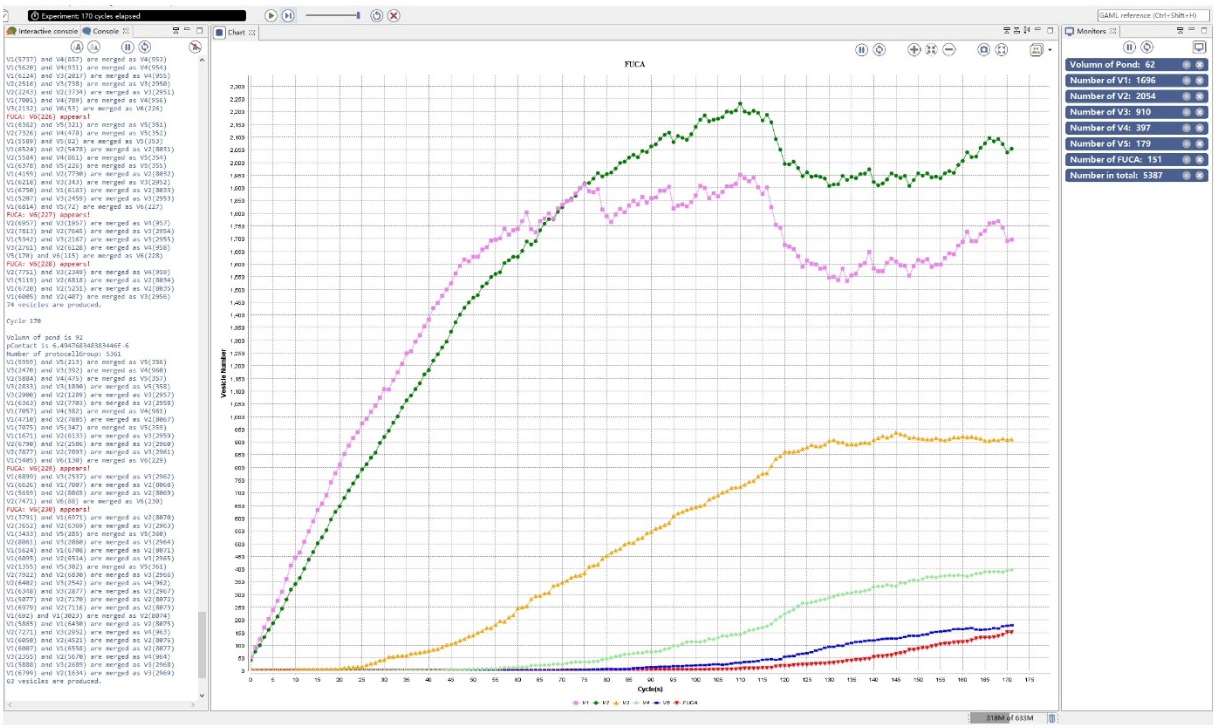

Figure S-1B. 70 FUCAs produced, 172 cycles

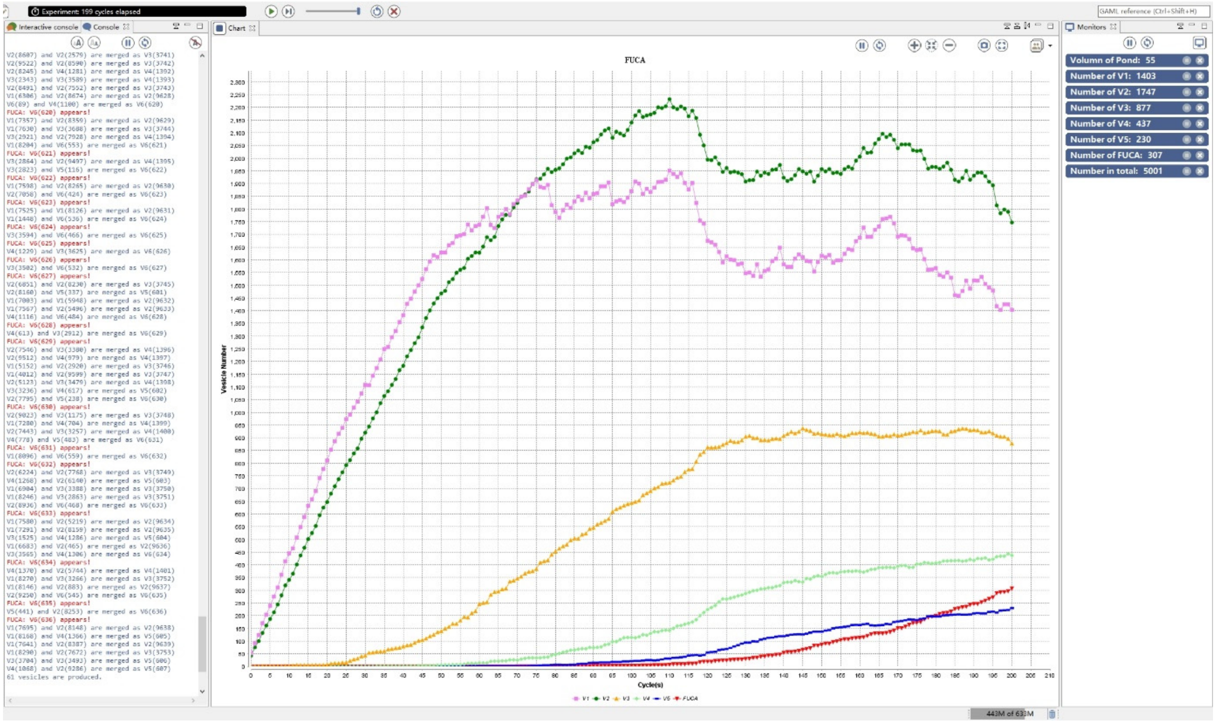

Figure S-1C: 139 FUCAs produced with 200 cycles

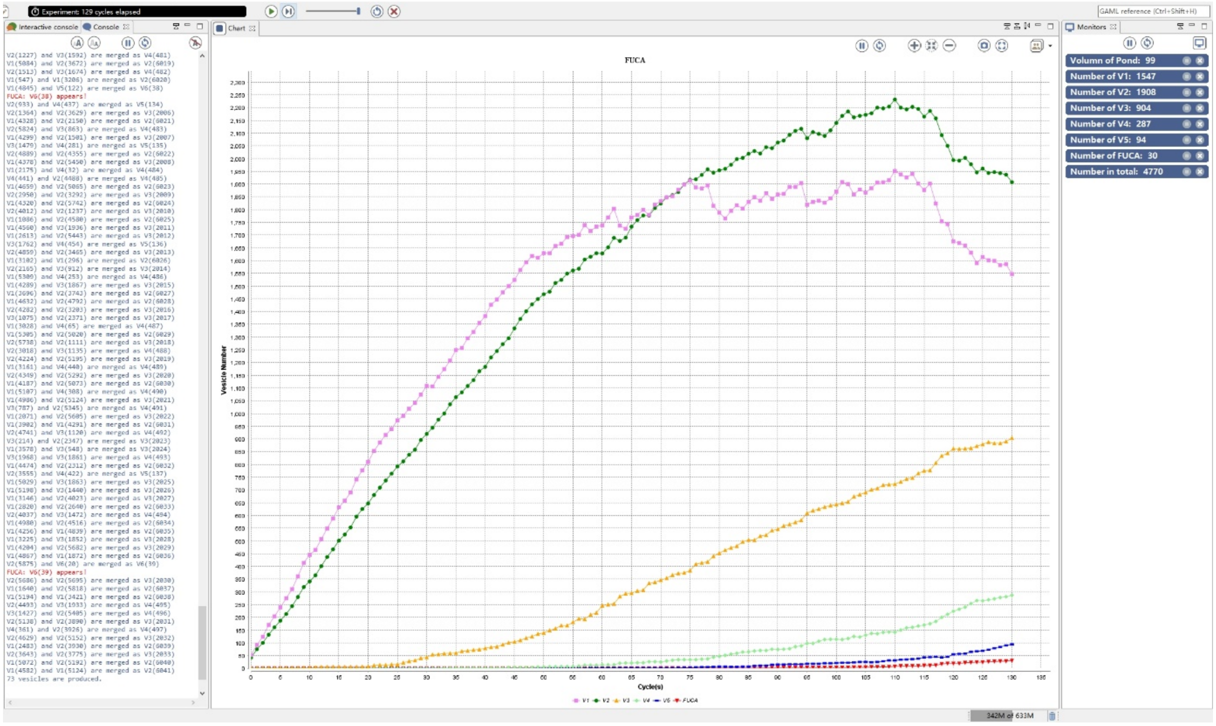

Figure S-1D: 284 FUCAs produced with 170 cycles

Figure S-2. Stage 2: Visualization of the Process of Synchronizing the SGC by two FUCAs. The left window of both panels records the evolutionary process of LUCA in simulation, with the red line highlights the appearance of the first LUCA with a full SGC. The right window plots the number of ticks ("cycles") for completing the synchronizing process, with its y-axis denoting the number of the sets of codons to be assigned. A simulation is halted when a LUCA emerges within it.

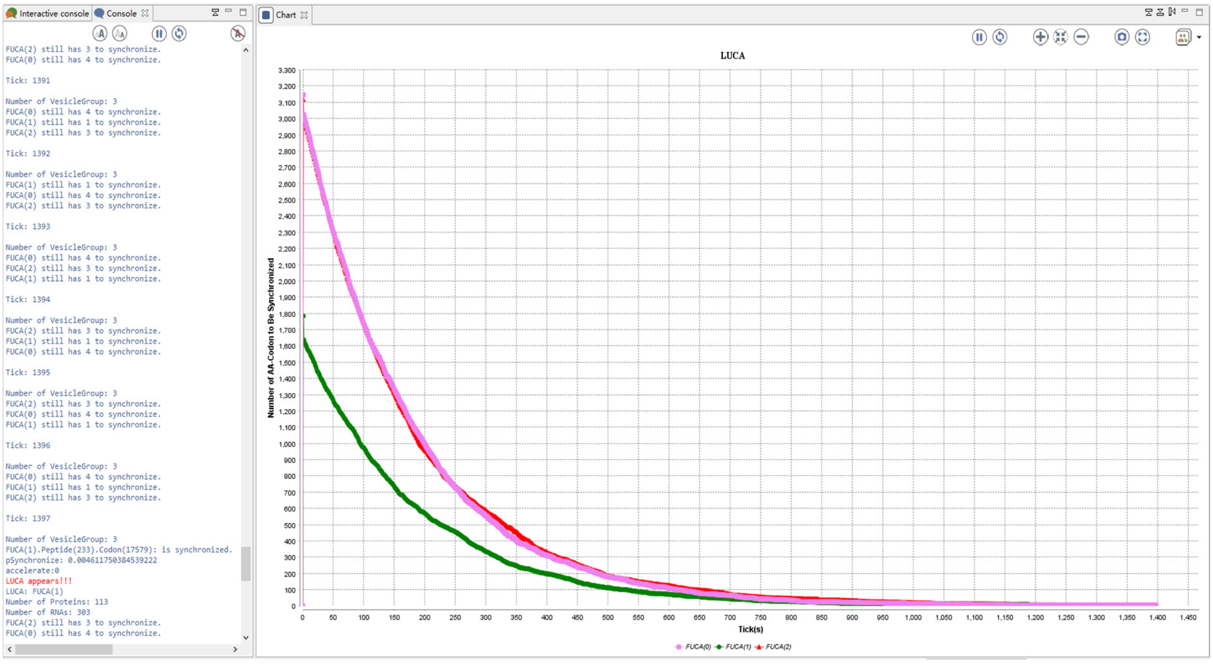

Fig. S2-A

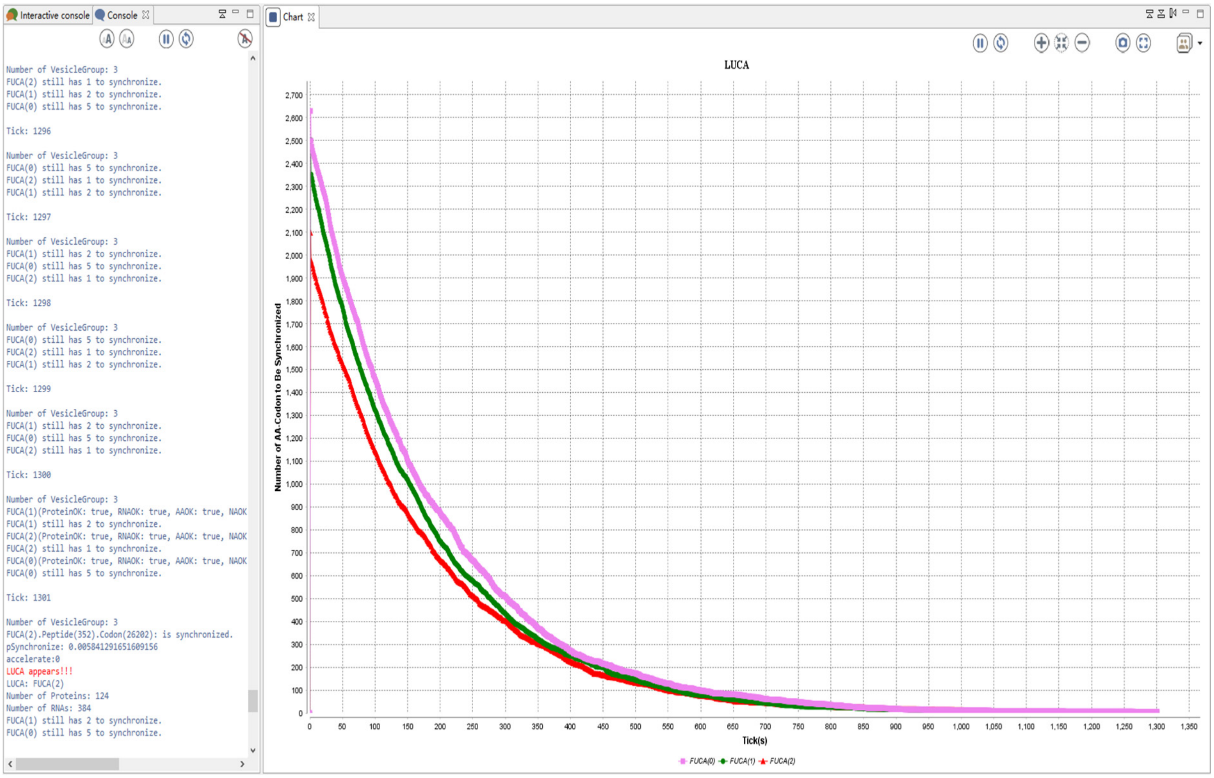

Fig. S2-B

Figure A-3. Snapshots, four simulations of the two stages together. Note: The left window of records the evolutionary process. The center window plots the number of “cycles” (x-axis) against the number of different types of vesicles (y-axis) within the pond as the system evolves. The right window summarizes key indicators, including the numbers of alive vesicles (V1-V6), of a specific simulation. When the first LUCA appears in a simulation run, the simulation is halted. See also Table 7 in the main document for details.

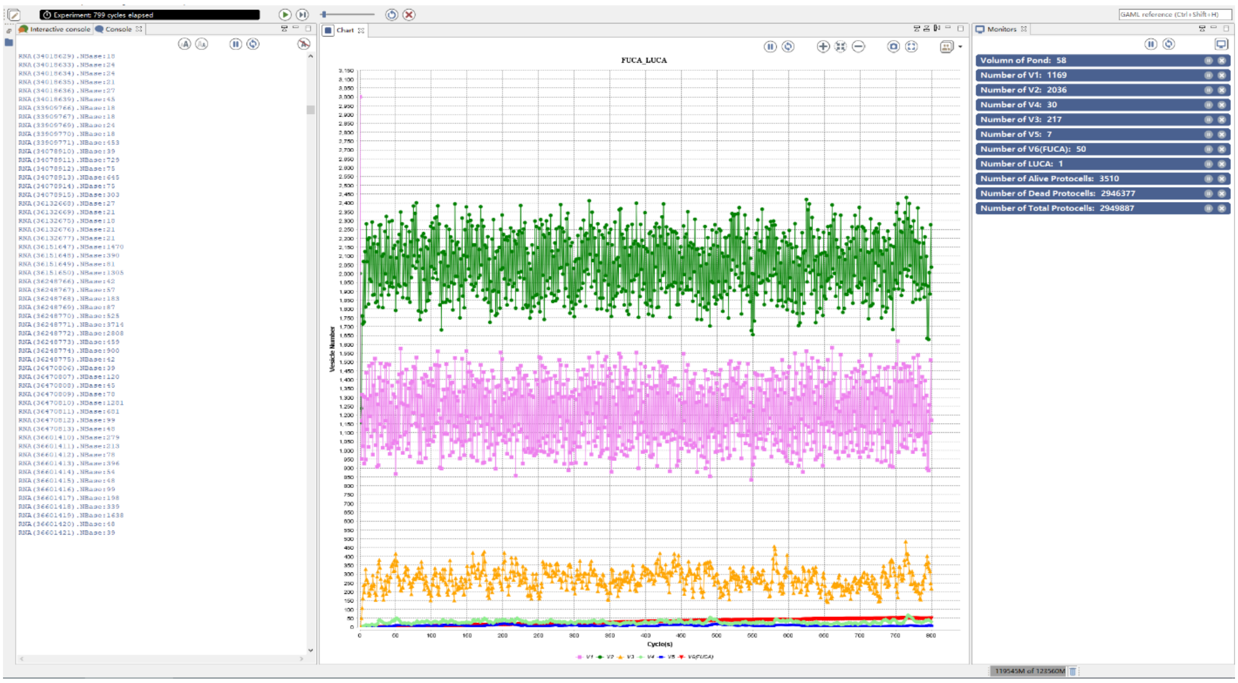

Fig. S-3A: 1st LUCA emerged with 800 cycles

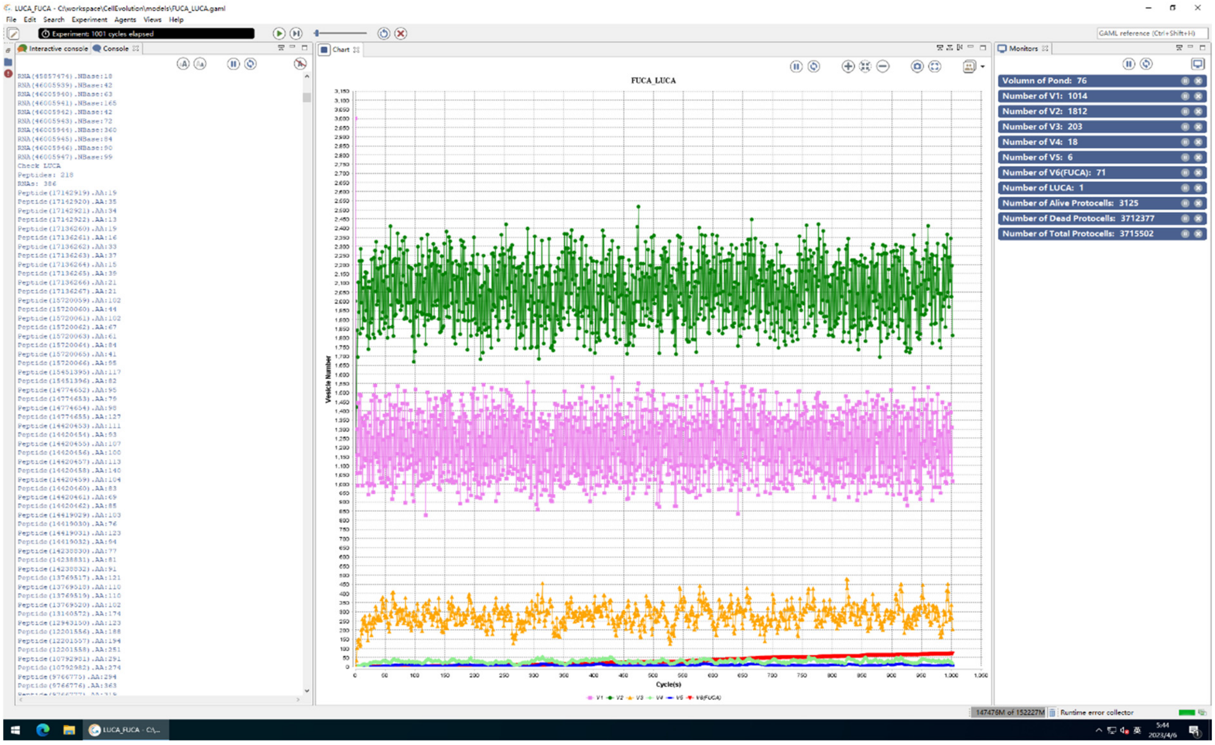

Fig. S-3B: 1st LUCA emerged with 1000 cycles

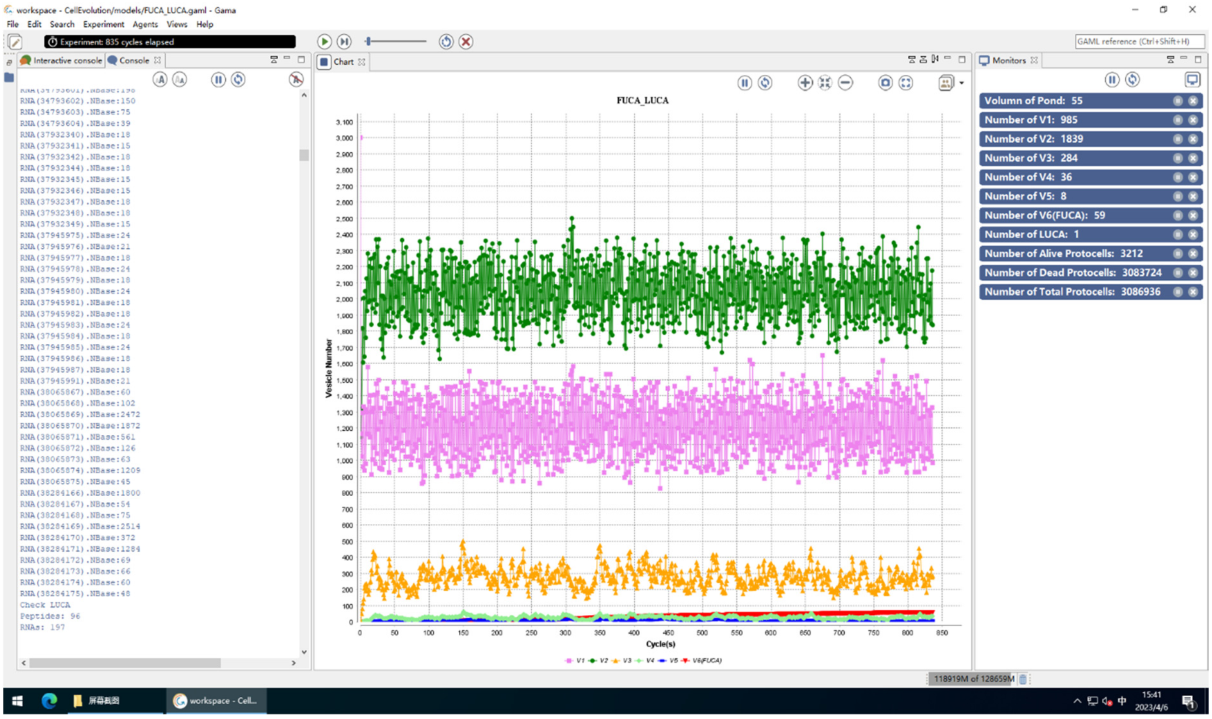

Fig. S-3C: 1st LUCA emerged with 840 cycles

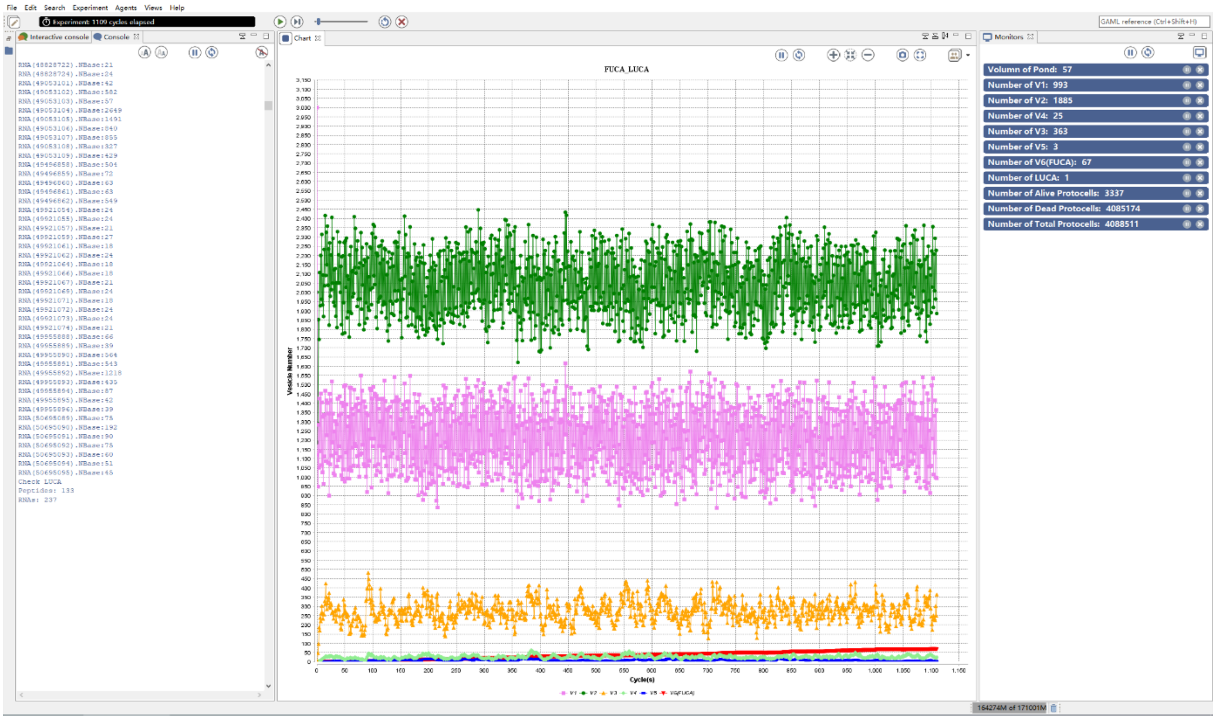

Fig. S-3D: 1st LUCA emerged with 1120 cycles

Figure S-4. Snapshots of four specific control simulations. Note: The left window of records the evolutionary process. The center window plots the number of "cycles" (x-axis) with the number of different types of vesicles (y-axis) within the pond as the system evolves. The right window summarizes key indicators, including the numbers of alive vesicles (V1-V6), of a specific simulation. As shown in these figures, when vesicles cannot merge with and acquire each other, no larger vesicles (i.e., V3 to V6) ever evolve in the system, and only V1 and V2 exist even though these control simulations were run with 1,320 to 1,440 cycles, which are at least 200 more cycles than the highest number of cycles (1,120) needed to produce LUCA in the positive simulations.
Please also compare Table 7 and Table 8 for details.

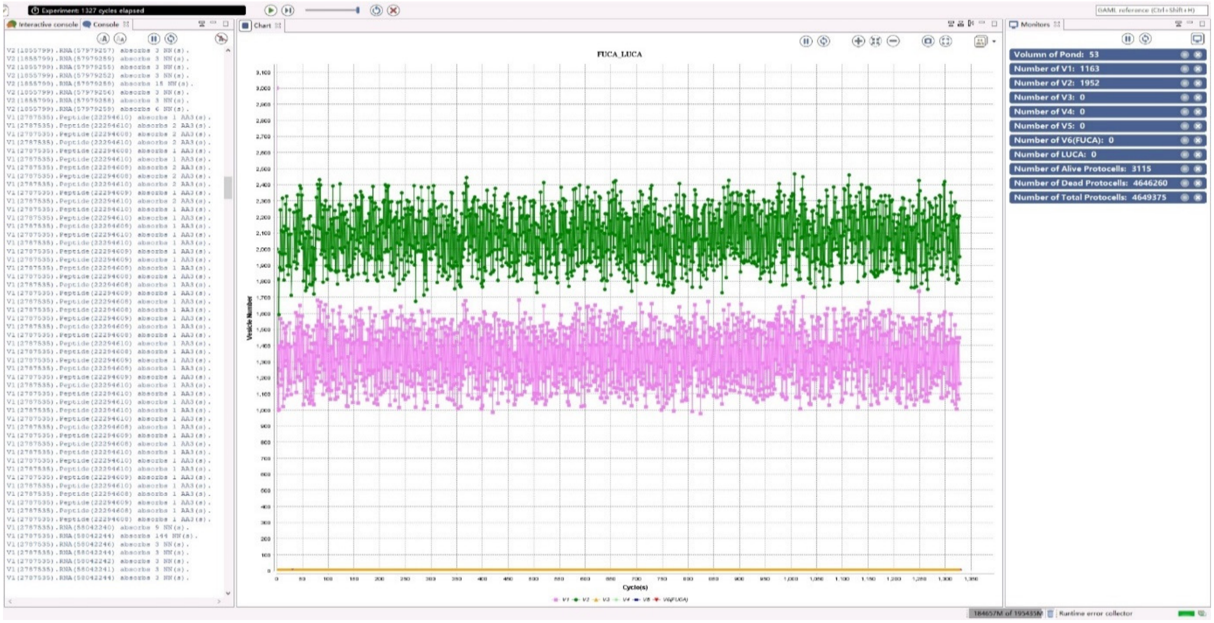

Fig. S-4A: no FUCA or LUCA produced with 1330 cycles

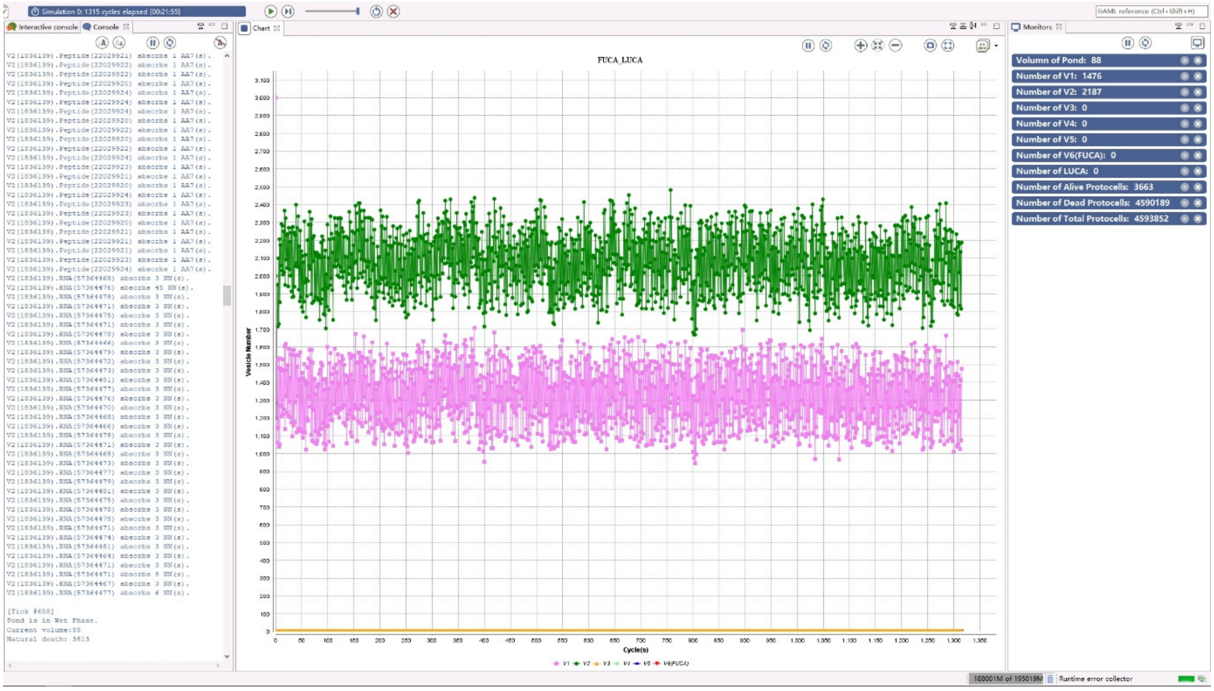

Fig. S-4B: no FUCA or LUCA produced with 1320 cycles

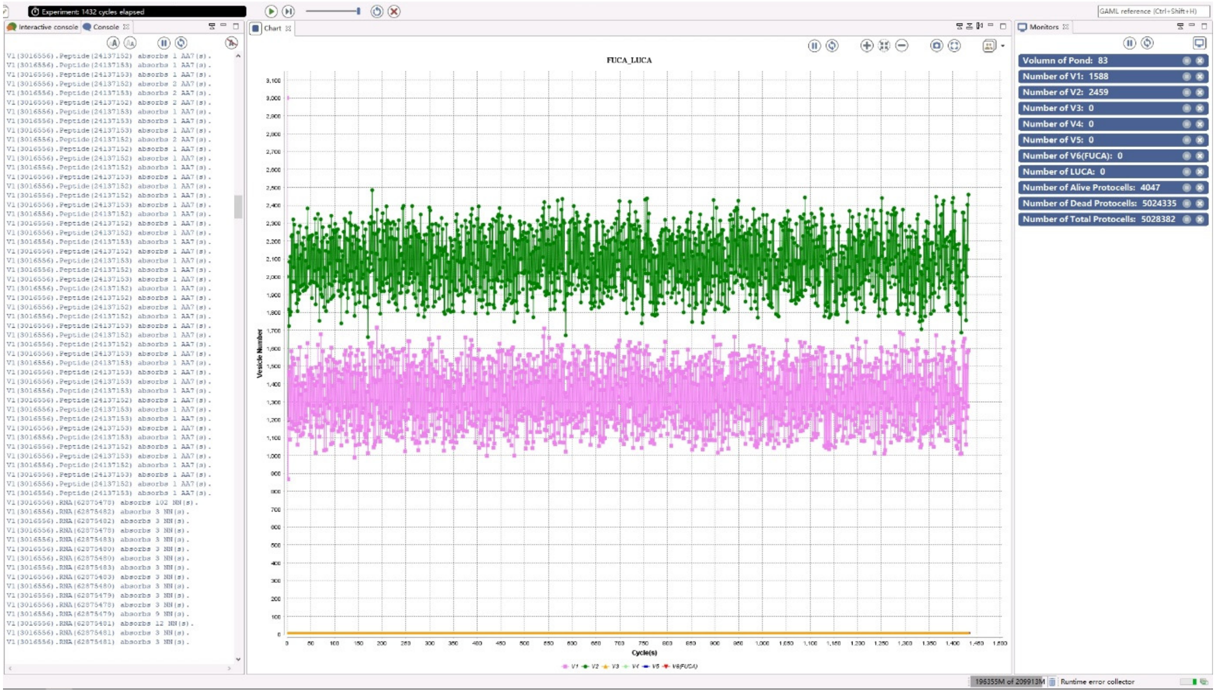

Fig. S-4C: no FUCA or LUCA produced with 1440 cycles

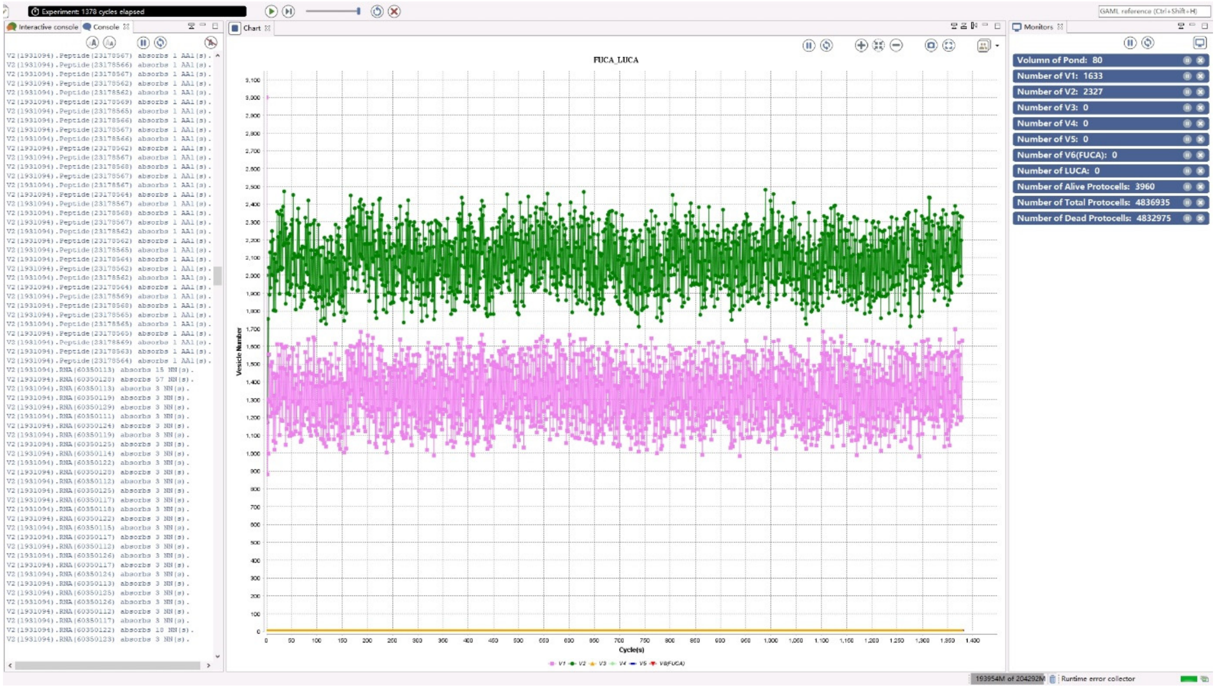

Fig. S-4D: no FUCA or LUCA produced with 1360 cycles
